# Does the physical activity profile change in patients with hip dysplasia from before to 1 year after periacetabular osteotomy?

**DOI:** 10.1080/17453674.2018.1531492

**Published:** 2018-10-18

**Authors:** Julie Sandell Jacobsen, Kristian Thorborg, Per Hölmich, Lars Bolvig, Stig Storgaard Jakobsen, Kjeld Søballe, Inger Mechlenburg

**Affiliations:** 1 Department of Physiotherapy and Department of Research in Rehabilitation and Health Promotion, Faculty of Health Sciences, VIA University College, Aarhus;;; 2 Department of Physiotherapy and Occupational Therapy, Aarhus University Hospital, Aarhus;;; 3 Sports Orthopaedic Research Center-Copenhagen (SORC-C), Department of Orthopaedic Surgery, Copenhagen University Hospital, Amager-Hvidovre, Hvidovre;;; 4 Department of Radiology, Aarhus University Hospital, Aarhus, Denmark;;; 5 Department of Orthopaedic Surgery, Aarhus University Hospital, Aarhus, Denmark;;; 6 Department of Clinical Medicine, Aarhus University, Aarhus, Denmark;;; 7 Department of Public Health, Aarhus University, Aarhus, Denmark

## Abstract

Background and purpose — Knowledge of physical activity profiles among patients with hip dysplasia is lacking. We investigated whether patients with hip dysplasia change physical activity profile from before to 1 year after periacetabular osteotomy. Furthermore, we investigated associations between change in accelerometer-based physical activity and change in self-reported participation in preferred physical activities (PA).

Patients and methods — Physical activity was objectively measured at very low to high intensity levels with accelerometer-based sensors. Subjectively, PA was recorded with Copenhagen Hip and Groin Outcome Score (HAGOS) in 77 patients. Associations between the 2 were analyzed with simple linear regression analyses.

Results — Changes in accelerometer-based physical activity ranged from –2.2 to 4.0% points at all intensity levels from baseline to 1-year follow-up. These changes represent very small effect sizes (–0.16 to 0.14). In contrast, self-reported PA showed a statistically and clinically relevant increase of 22 (CI 14–29) HAGOS PA points 1 year post-surgery. Associations between change in accelerometer-based physical activity and change in self-reported PA were, however, not statistically significant and correspond to a percentage change in physical activity of only –0.87% to 0.65% for a change of 10 HAGOS PA points.

Interpretation — Patients with hip dysplasia do not seem to change physical activity profile 1 year post-surgery if measured with objective accelerometer-based sensors. This is interesting as self-reported PA indicates that patients’ ability to participate in physical activity increases, suggesting that this increased self-reported participatory capacity is not manifested as increased objectively measured physical activity.

Hip dysplasia is a pathological development of the hip joint, presenting in early adulthood. The clinical presentation of symptomatic hip dysplasia is pain caused by intra-articular injury (Nunley et al. 2011) and coexisting muscle–tendon pain (Jacobsen et al. 2018b). The pain prevents patients from participating in their preferred physical activities (Jacobsen et al. 2013), including higher intensity activities such as running (Novais et al. 2013).

Periacetabular osteotomy (PAO) is the preferred joint-preserving treatment for patients with symptomatic hip dysplasia (Troelsen et al. 2008). PAO has been shown to result in pain relief, restoration of function, and high hip survival rates (Lerch et al. 2017). Furthermore, previous studies on self-reported physical activity have shown that patients increase their ability to participate in their preferred physical activities 1 year after PAO (Jacobsen et al. 2014, Klit et al. 2014, Khan et al. 2017) and spend more time on physical activities at higher intensities (Novais et al. 2013). Discrepancies between self-reported physical activity and objectively measured physical activity have been reported (Sallis and Saelens 2000, Harding et al. 2014). However, it is important to recognize that self-reported physical activity captures the capability to complete a given task, while the ability to measure the actual performance is limited (Smuck et al. 2017). Accelerometer-based measures of physical activity allow us to evaluate if patients with hip dysplasia change objectively measured physical activity profile, alongside with subjective measured levels of improved pain, physical function, and quality of life after PAO.

We investigated whether patients with hip dysplasia change physical activity profile from before to 1 year after periacetabular osteotomy, measured by accelerometer-based sensors and self-reported physical activity. Furthermore, we investigated associations between change in accelerometer-based physical activity and change in self-reported participation in preferred physical activities (PA).

## Patients and methods

### Patients

100 consecutive patients with unilateral and bilateral symptomatic hip dysplasia were included in this study from May 2014 to August 2015 at the Department of Orthopedics, Aarhus University Hospital, Denmark. Inclusion criteria to participate in this study was Wiberg’s center-edge (CE) angle <25 degrees (Wiberg 1939), groin pain for at least 3 months, and scheduled PAO. Patients with known co-morbidities and a history of previous surgical interventions affecting the hip function were excluded. Further details on study design was reported in our previous studies on the same study population, reporting ultrasonography-detected abnormalities and prevalence of muscle–tendon pain prior to PAO (Jacobsen et al. 2018a, b).

### Baseline characteristics

Baseline characteristics were recorded at baseline by standardized questions. Pain at rest was measured on a numeric rating scale (NRS), and radiographic acetabular angles and Tönnis’s osteoarthritis grade were measured by a single rater using standing anteroposterior radiographs. Hospital charts were used to retrieve data on previous surgeries and co-morbidities.

### Periacetabular osteotomy

2 experienced orthopedic surgeons used the minimally invasive approach for PAO. The surgical procedure involves reorientation of the acetabulum through 3 separate osteotomies and correction of the insufficient coverage of the femoral head. Compared with other PAO procedures, the minimally invasive approach involves minor incision of the soft tissue and enables early mobilization (Troelsen et al. 2008). Post-surgery, all patients underwent an in-hospital standardized rehabilitation program, and only partial weight bearing with a maximum load of 30 kg was allowed in the first 6–8 weeks. Patients were discharged after approximately 2 days.

### Accelerometer-based physical activity

The patients’ habitual physical activity profile was objectively measured with a commercial tri-axial accelerometer-based sensor (Gulf Coast Data Concepts, Waveland, MS, USA) during 7 consecutive days (working and leisure days) at baseline and 1 year after PAO. The sensor was taped to the lateral non-affected upper leg of patients using hypo-allergenic double-sided tape (3M, Maplewood, MN, USA). The position of the sensor was at the mid-thigh between the trochanter and lateral condyle, with the y-axis of the sensor along the axis of the femur (van Laarhoven et al. 2016). The sensor was worn during waking hours for a minimum of 8 hours per day. It was removed for the night and during both showering and swimming activities. Data from –6g to 6g were sampled at 50 Hz.

The raw activity data were transferred to a computer and processed using a validated algorithm (Lipperts et al. 2017). Data were visually divided into separate days using a MatLab (https://www.mathworks.com/products/matlab.html) script designed for the purpose, and non-worn days were excluded. All data were calibrated manually by selecting a period of level walking in the dataset of each day to adapt variations in height, morphology, sensor placement, walking style, and speed (Lipperts et al. 2017). After calibration, the data were run through an algorithm based on a decision tree, and divided into resting, standing, cycling, level walking, walking on stairs (upstairs and downstairs), and running. Further details are described by Lipperts et al. (2017). In addition, the algorithm constructed an intensity variable on the distribution of the average signal intensity in 10-second intervals grouped into 4 intensity levels. The intensity levels were: (i) Very low intensity such as sitting and standing (0–0.05g), (ii) Low intensity such as standing and shuffling (0.05–0.1g), (iii) Moderate intensity such as slow and normal walking (0.1–0.2g), (iv) High intensity such as brisk walking, running, and jumping (> 0.2g).

### Self-reported physical activity

Self-reported physical activity was subjectively measured as preferred physical activity participation (PA) using the Copenhagen Hip and Groin Outcome Score (HAGOS) (Thorborg et al. 2011), and completed by the patients at baseline and 1 year after PAO. Besides participation in physical activities, the HAGOS outcome score captures pain, symptoms, physical function in daily living (ADL), physical function in sports and recreation, and quality of life on a scale from 0 to 100 points, where 100 points indicate the highest outcome. HAGOS is a valid, reliable, and responsive outcome measure developed for young patients with longstanding hip and groin pain (Thorborg et al. 2011). Additionally, time spent on preferred PA per week was recorded at baseline and 1 year after PAO.

### Sample size consideration

The purpose of this study was to investigate whether patients change physical activity profile from before to 1 year after surgery. We considered moderate effect sizes to be of importance, which required 85 patients for detecting an effect size of 0.5 from before to 1 year after surgery. This calculation was based on a power of 90% and a significance level of 5%.

### Statistics

Parametric continuous outcome measures were reported as means (SD) if normally distributed; otherwise reported as medians with either interquartile ranges (IQR) or 95% confidence intervals (CI). Normality was assessed with histograms and probability plots. Categorical data were reported as number of events and percentage with CI. Time spent on physical activities was normalized to total accelerometer-based wear time before surgery and 1 year after surgery. Number of events at baseline were normalized to average daily accelerometer-based wear time at baseline, and the number of events at 1-year follow-up were normalized to average daily accelerometer-based wear time at 1-year follow-up. Differences between baseline and 1-year follow-up were tested with a paired t-test if assumptions were met and otherwise tested with the non-parametric Wilcoxon signed-rank test. Effect sizes were calculated from the paired t-test as Cohen’s d on the formula: t statistic/√(n) for parametric data and otherwise from the non-parametric Wilcoxon signed-rank test on the formula: z statistic/√(n). Furthermore, simple linear regression analyses were performed assessing associations between change in objectively measured physical activity at 4 intensity levels (very low, low, moderate, and high intensity) and change in subjectively measured HAGOS PA from baseline to 1 year post-surgery. Crude ß-coefficients were estimated, and the assumptions (independent observations, linear association, constant variance of residuals, and normal distribution of residuals) for the linear regression analyses were met. The crude ß-coefficients refer to the slope of the regression line (the increase in objectively measured physical activity for every unit increase in subjectively measured HAGOS PA). STATA 14.0 (StataCorp, College Station, TX, USA) software package was used for data analysis and significant results were presented if p < 0.05.

### Ethics, registration, funding, and potential conflict of interests

This study was done in accordance with the STROBE statement and in compliance with the Helsinki Declaration. The study was notified to the Central Denmark Region Committee on Biomedical Research Ethics on January 14, 2014 (5/2014). The Danish Data Protection Agency gave permission for the handling of personal data (1-16-02-47-14), and the protocol was registered at ClinicalTrials.gov (20140401PAO). All patients gave informed written consent. This study was funded by the Danish Rheumatism Association [grant number: A3280], the Aase and Ejnar Danielsen Fund [grant number: 10-000761/LPJ], and the Fund of Family Kjaersgaard, Sunds [date: 01-05-2018]. The authors have no competing interests.

## Results

**Figure 1.**
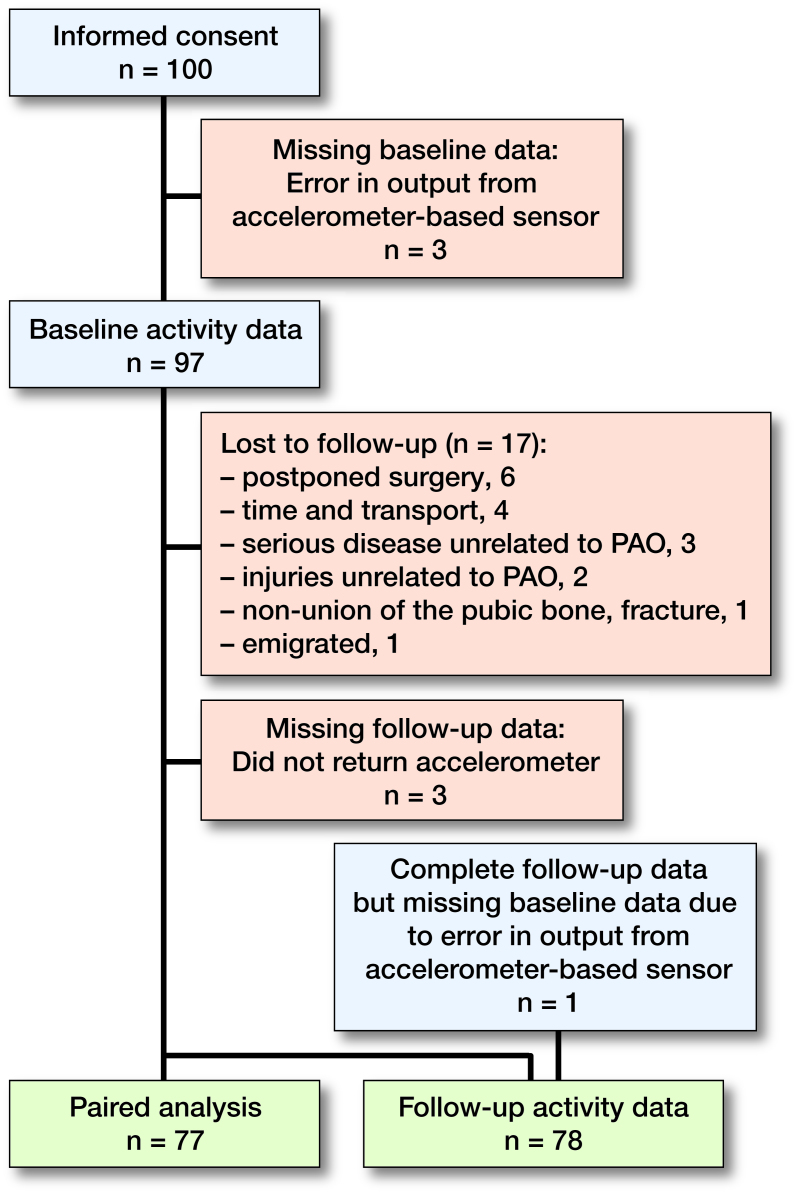
Flowchart of the study process among 100 consecutive patients with symptomatic hip dysplasia scheduled for periacetabular osteotomy (PAO).

100 consecutively enrolled patients gave informed consent and were included in this study. Data on objective accelerometer-based physical activity were lost on 3 patients at baseline. At 1-year follow-up 17 patients were lost to follow-up and objective accelerometer-based physical activity data were lost on further 3 patients (Figure 1).

Baseline characteristics of the included patient are reported in Table 1. Pain at rest measured on an NRS was 3.0 (1.5–4.0) at baseline, and at 1-year follow-up it was 0.0 (0.0–1.3). A sensitivity analysis of the 17 patients lost to follow-up revealed no differences in age, sex, pain, and radiological angles compared with the analyzed patients.

**Table 1.** Baseline characteristics of 97 consecutive patients with hip dysplasia

Outcomes	Baseline
Men	15
Age, years (SD)	30 (9)
BMI, kg/m^2^ (SD)	23 (3)
CE angle, degrees (SD)	17 (4.7)
AI angle, degrees (SD)	14 (4.8)
Tönnis osteoarthritis grade 1	3
Bilateral hip dysplasia	86
Hours spent on self-reported preferred	
physical activities/week, n	
<2.5	13
2.5 to <5	18
5 to <10	40
≥10	26
Self-reported preferred physical activities, n	
Fitness	27
Running	19
Team sports	13
Gymnastics	6
Horseback riding	6
Bicycling	5
Dancing	3
Other	16
No one	2

Abbreviations: SD (standard deviation); BMI (body mass index), CE (center-edge), AI (Tönnis’ acetabular index).

### Accelerometer-based physical activity

The accelerometer-based sensor was worn for a median of 7 days (3–8) at both baseline and 1-year follow-up; only 6 patients had worn the sensor for less than 5 days. Changes in objectively measured physical activity ranging from –2.2 to 4.0% points, across all intensity levels (covering very low, low, moderate, and high intensity) from baseline to 1-year follow-up (Table 2). This was, however, not statistically significant, and represents very small effect sizes ranging from –0.16 to 0.14. A general physical activity profile of the patients is reported in Table 3.

**Table 2.** Accelerometer-based physical activity and self-reported physical activity in hip dysplasia (n = 77)

Outcomes	Baseline mean (SD)	1-year follow-up mean (SD)	Difference mean % points (CI)	Effect size	p-value
Percent of time measured by accelerometer-based sensors					
Very low intensity	74 (9.1)	76 (8.9)	1.6 (–0.89 to 4.0)	0.14	0.2
Low intensity	14 (5.1)	14 (5.3)	–0.66 (–2.2 to 0.89)	–0.096	0.4
Moderate intensity	6.8 (2.9)	6.4 (2.6)	–0.40 (–1.1 to 0.31)	–0.13	0.3
High intensity	4.6 (2.0)	4.3 (2.0)	–0.32 (–0.77 to 0.13)	–0.16	0.2
Self-reported physical activity					
HAGOS PA^a^	23 (24)	45 (33)	22 (14 to 29)	0.67	<0.001
Hours in PA/week^b^,^c^	0.0 (0.0–2.3)	1.0 (0.0–3.0)	–	0.18	0.1

a HAGOS (Copenhagen Hip and Groin Outcome Score, 0–100 points),

b PA (preferred physical activity participation)

c Non-parametric data presented as median (interquartile range).

**Table 3.** General physical activity profile based on objective data on patients with hip dysplasia

Outcomes	Baseline (n = 97) median (CI)	1-year follow-up (n = 78) median (CI)
Cadence as steps/min	99 (98–100)	100 (98–102)
Events/day, n		
Total steps	7,404 (6,645–8,418)	7,925 (6,637–8,612)
Steps (level)	6,923 (6,192–7,709)	7,322 (6,081–8,217)
Steps (up)	266 (194–403)	235 (171–313)
Steps (down)	155 (134–183)	146 (123–169)
Total wear time, h/day	14 (14–15)	15 (14–15)
Time as percentage		
Resting	64 (61–68)	63 (59–66)
Standing	23 (22–27)	26 (23–27)
Walking	11 (9.9–13)	11 (9.3–13)
Cycling	0.15 (0.063–0.33)	0.084 (0.046–0.18)
Running	0.011 (0.0042–0.020)	0.0078 (0.0040–0.025)

### Self-reported physical activity

Subjectively measured HAGOS PA increased statistically significantly from baseline to 1-year follow-up with a moderate effect size. Similarly, a statistically non-significant change in hours spent on preferred physical activities at 1-year follow-up was present, represented by a very small effect size (Table 2).

### Associations between accelerometer-based physical activity and self-reported physical activity

**Figure 2.**
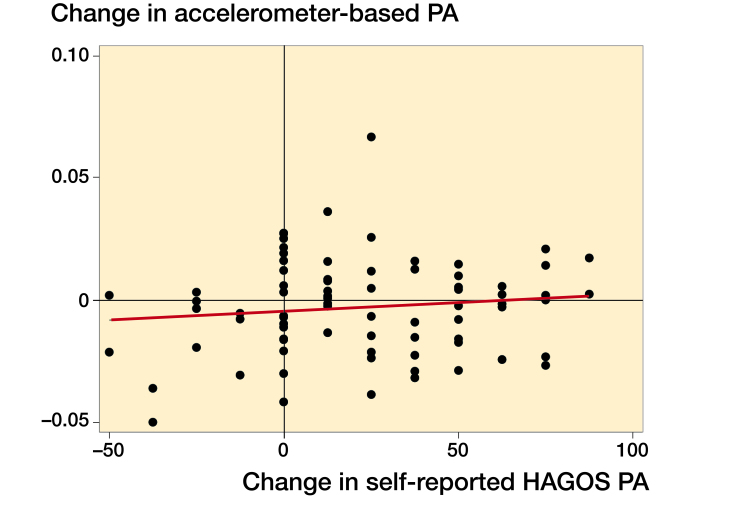
Scatter plot of the prediction of change in accelerometer-based physical activity (PA) at high intensity as a linear function of change in self-reported preferred physical activity participation (PA) measured by the Copenhagen Hip and Groin Outcome Score (HAGOS) from baseline to 1 year post-surgery.

Associations between change in accelerometer-based physical activity and change in HAGOS PA ranged from –0.00087 to 0.00065, covering very low, low, moderate, and high intensity from baseline to 1-year follow-up. These were, however, not statistically significant and correspond to a percentage change in physical activity of only –0.87% to 0.65% for a change of 10 HAGOS PA points. The individual associations were as follows: Very low intensity: ß = –0.00011 (CI –0.00087 to 0.00065), p = 0.8. Low intensity: ß = –0.000022 (CI –0.00050 to 0.00046), p = 0.9. Moderate intensity: ß = 0.000044 (CI –0.00018 to 0.00027), p = 0.7. High intensity: ß = 0.000072 (CI –0.000066 to 0.00021), p = 0.3 (Figure 2).

## Discussion

Using accelerometer-based sensors, we found that the objectively measured physical activity profile was the same before and 1 year after PAO at all the measured physical activity intensity levels, indicated by very low effect sizes. In contrast, subjectively measured physical activity showed a moderate increase, and this was higher than the minimal important change (MIC) of HAGOS PA (Thomeé et al. 2014). Our linear regression analyses showed absence of any statistically significant and clinically relevant associations between objectively and subjectively measured physical activity as a change of 10 HAGOS PA points would maximally result in a change of physical activity of 1%.

In a systematic review, Tudor-Locke et al. (2011) reported minimum international recommendations for physical activity in healthy populations. The minimum number of steps for adults was 7–8,000 steps per day with an average cadence of 100 steps per minute indicating moderate intensity walking (Tudor-Locke et al. 2011). The general physical profile (Table 3) of our patients shows that their overall level of physical activity was at level with reference values for both steps and cadence at baseline and at the 1-year follow-up, indicating that the patients followed current public health guidelines. Similarly, an American study reported daily strides by a step watch in adults with hip dysplasia and in healthy adults. No differences were seen between the 2 groups (Harris-Hayes et al. 2012). The average number of strides per day was 4,627 (i.e., 9,254 steps) among the adults with hip dysplasia, similar to the average number of steps reported among our patients. Additionally, physical activity was grouped into 4 different intensity levels based on strides per minute. No differences between the groups were found, and the effect sizes were very low (Harris-Hayes et al. 2012). Overall, these 4 intensity levels were comparable to the 4 intensity levels for physical activity in our study, and the results were similar to our results. This suggests that the physical activity profile among Danish patients with hip dysplasia is comparable to that of healthy American adults both before and 1 year after PAO, when measured objectively with accelerometer-based sensors.

This finding is in line with observations among patients undergoing total hip arthroplasty. A previous study measured physical activity with objective accelerometer-based sensors in 19 patients (mean age 69 years) with hip osteoarthritis before and 6 months after total hip arthroplasty (Harding et al. 2014). Similar to our findings, no significant increase in objectively measured physical activity was found after surgery. Self-reported physical activity after surgery has been reported in 6 previous studies of patients with hip dysplasia (Novais et al. 2013, Jacobsen et al. 2014, Beaulé et al. 2015, Clohisy et al. 2017, Hara et al. 2017, Khan et al. 2017). In 1 prospective, multicenter study, the physical activity level was measured subjectively with the University of California, Los Angeles (UCLA) activity scale in 371 patients (mean age 25 years) with hip dysplasia (Clohisy et al. 2017). The patients’ physical activity level had increased statistically significantly 2 years post-surgery, alongside with improvement in self-reported pain, physical function, and quality of life. These results are in line with the results of 4 other previous studies in patients with hip dysplasia reporting statistically significantly and clinically relevant increases in self-reported physical activity levels after surgery (Novais et al. 2013, Beaulé et al. 2015, Hara et al. 2017, Khan et al. 2017). In a 6th study, a statistically significantly and clinically relevant increase in HAGOS PA was reported among 32 patients (mean age 34 years) with hip dysplasia 1 year after PAO compared with baseline (Jacobsen et al. 2014). Overall, the results of the 6 previous studies showed statistically significantly and clinically relevant increases in subjectively measured physical activity levels after surgery (Novais et al. 2013, Jacobsen et al. 2014, Beaulé et al. 2015, Clohisy et al. 2017, Hara et al. 2017, Khan et al. 2017); this corroborates our findings.

Difficulties in participating in preferred physical activities are reported as important components of disability among patients with hip pain (Novais et al. 2013). We observed increased HAGOS PA and unchanged objectively measured physical activity 1 year post-surgery. The discrepancy probably exists because HAGOS PA and accelerometer-based methods measure different aspects of physical activity. Accelerometer-based methods provide an objective measure of physical activity, while HAGOS PA measures the patients’ self-perceived ability to participate in preferred physical activity. Moreover, age, employment status, culture, and geography have been shown to be associated with activity level (Hirata et al. 2006, Sechriest II et al. 2007, Althoff et al. 2017). Changing one’s physical activity profile is complex (Harding et al. 2014), because it is under the influence of one’s lifestyle and thus both patients and health professionals need to be aware that PAO alone is unlikely to change the physical activity profile from before to 1 year after PAO.

### Limitations

We included no healthy controls and estimates of both accelerometer-based physical activity and self-reported physical activity could therefore not be compared with estimates in healthy controls. The negative impact of this is presumably small since it was possible to compare our findings with the findings of a previous study that included a control group (Jacobsen et al. 2014). Processing of the accelerometer-based physical activity data does involve some measure of subjectivity as the researcher has to calibrate the algorithm manually (Lipperts et al. 2017). To reduce potentially systematic effects, we processed all data in pairs, and in this way processing of baseline data was followed by processing of 1-year follow-up data in each patient. Still, we cannot rule out that the subjective element may increase the overall variability. The intensity

variable shows the percentage of time at 4 different intensity levels, and other thresholds may have given other results. However, a previous study on young and middle-aged adults with hip disorders found similar results, even though their intensity levels were based on strides per minute (Harris-Hayes et al. 2012). This similarity suggests that our findings are valid.

### Summary

Patients with hip dysplasia do not seem to change physical activity profile 1 year post-surgery if measured with objective accelerometer-based sensors. This is interesting as self-reported PA indicates that patients’ ability to participate in physical activity increases, suggesting that this increased participatory capacity is not manifested as increased objectively measured physical activity. These findings are similar to another study on patients undergoing total hip arthroplasty where no increase in accelerometer-based physical activity was found after surgery.

All authors took part in planning the study and writing of the present manuscript. IM, KT, PH, and LB concentrated on the design and use of methods. JSJ tested the participants. KS and SSJ were responsible for inclusion of patients, and SSJ made the radiologic evaluations. JSJ coordinated all elements and made the first draft of the manuscript.

The authors would like to thank Bernd Grimm, Matthijs Lipperts, and Marianne Tjur for their invaluable help and advice carrying out the accelerometer analysis. Furthermore, they would like to thank Charlotte Møller Sørensen and Gitte Hjørnholm Madsen for their assistance in collecting accelerometer data from the patients.

*Acta* thanks Jan Erik Madsen and other anonymous reviewers for help with peer review of this study.
